# Flooding, season and habitat interact to drive changes in vertebrate scavenging and carcass persistence rates

**DOI:** 10.1007/s00442-024-05531-0

**Published:** 2024-04-08

**Authors:** Zyna Krige, Emma E. Spencer, Mathew S. Crowther, Christopher R. Dickman, Thomas M. Newsome

**Affiliations:** The University of Sydney, Sydney, NSW Australia

**Keywords:** Carcass, Carrion, Desert, Flood, Vertebrates

## Abstract

Scavenging dynamics are influenced by many abiotic and biotic factors, but there is little knowledge of how scavengers respond to extreme weather events. As carrion is a major driver of the organisation and structure of food webs within ecological communities, understanding the response of scavengers to extreme weather events is critical in a world that is increasingly subject to climate change. In this study, vertebrate scavenging and carcass persistence rates were quantified in the Simpson Desert of central Australia; a system that experiences major fluctuations and extremes in weather conditions. Specifically, a total of 80 adult red kangaroo (*Osphranter rufus*) carcasses were placed on the landscape and monitored using remote sensor cameras. This included 40 carcasses monitored before and then 40 carcasses monitored after a major flooding event. The carcasses were monitored equally before and after the flood across different seasons (warm and cool) and in dune and interdune habitats. Overall, a total of 8124 scavenging events for 97,976 visitation minutes were recorded for 11 vertebrate species within 30 days of carcass placement pre- and post-flood. Vertebrate scavenging increased post-flood in the warm season, especially by corvids which quadrupled their scavenging events during this time. There was little difference in carcass persistence between habitats, but carcasses persisted 5.3-fold longer post-flood in warm seasons despite increased vertebrate scavenging. The results demonstrate that a flood event can influence scavenging dynamics and suggest a need to further understand how seasons, habitats and extreme weather events can drive changes in carrion-based food webs.

**Supplementary Information:**

The online version contains supplementary material available at 10.1007/s00442-024-05531-0.

## Introduction

The distribution of food resources is a major determinant of the abundance, behaviour and assemblage structure of consumer organisms (Letnic and Dickman 2010). However, different types of food resources influence consumers and ecosystems in different ways. For example, highly localised and ephemeral resources such as carrion support and attract a diverse range of microbial, invertebrate and vertebrate scavenger species (DeVault et al. 2003; Barton et al. 2013). The high-quality food represented by carrion is important in attracting these varied scavengers, which in turn contribute to the recycling of energy and nutrients throughout ecosystems (Wilson and Wolkovich 2011; Barton et al. 2013). As a hotspot for biogeochemical activity, the interactions that take place around carrion and the subsequent availability of carrion resources can drive scavenging community structure, activity and functioning in ecosystems (DeVault et al. 2003; Barton et al. 2013; Benbow et al. 2019). Carrion thus provides a unique focal location to quantify the effects of resource distribution and availability on the organisation of scavenger communities and food webs more broadly.

At any given carcass site, the composition of scavenger guilds can affect carcass persistence rates and the subsequent activity of other scavengers (Moleón et al. 2014). For example, large and dominant (apex) scavengers including vultures and wolves (*Canis* spp.) can rapidly consume large carcasses, effectively modulating carrion distribution and limiting scavenging by smaller and subordinate (meso) species through resource monopolisation and competitive exclusion (Pereira et al. 2014; Allen et al. 2014; Morales-Reyes et al. 2017). However, meso-scavengers may also be highly efficient in detecting and consuming carrion, with species such as ravens (*Corvus* spp.) and red foxes (*Vulpes vulpes*) contributing significantly to carcass biomass removal in some cases (Selva et al. 2005; Brown et al. 2015). In this way, the relative use of carcasses by different vertebrate scavengers can affect the period over which carcasses are available, as well as resultant rates of scavenging by other species.

The distribution of carrion and the extent of vertebrate scavenging are also affected by myriad other factors. For example, climatic conditions including seasonality and temperature have profound effects on decomposition processes, with microbial and insect activity typically increasing in warm conditions and thus accelerating rates of carcass decay (DeVault et al. 2003; Carter et al. 2008; Barton et al. 2013). Longer carcass persistence times typically occur in seasons or areas with lower temperatures, with vertebrate scavengers also responding to changes in seasonal conditions—although the direction of response by vertebrate scavengers varies (DeVault and Rhodes 2002; DeVault et al. 2004; Pereira et al. 2014; Turner et al. 2017). Scavenging efficiency, in terms of carcass detection, is also affected by the structural characteristics of the environment such as vegetation and canopy cover (Turner et al. 2017; Pardo-Barquín et al. 2019). Avian scavengers that rely predominantly on sight to locate food often find and utilise carcasses in open and clear areas due to ease of accessibility and detection, while the opposite occurs for ground-based scavengers that frequent carcasses in more densely vegetated areas, highlighting distinct differences in scavenger assemblage composition between contrasting habitat types (Carrasco-Garcia et al. 2018). Finally, food availability can influence rates of scavenging such that scavengers may switch from predation to scavenging if prey is in decline or if there are excess carcass loads following mass die off events (Parsons et al. 2022).

The impact of abiotic and biotic factors on scavenging dynamics and carcass persistence has been studied extensively in northern hemisphere systems (Selva et al. 2005; Turner et al. 2017; Pardo-Barquín et al. 2019). But previous studies have generally assessed small-scale changes in scavenging dynamics or carcass persistence across different seasons and habitats. On the other hand, much less is known about the influence that large-scale disturbance events have on scavenging dynamics and decomposition. However, Newsome and Spencer (2022) found that avian scavengers detected carcasses faster in open than closed habitats and following a widespread fire event. This demonstrated that habitat effects coupled with an extensive disturbance event can influence scavenging dynamics, leading to the question of how scavengers might respond to other types of major environmental disturbances.

Arid environments are strongly dominated by extrinsic environmental forces, particularly rainfall and temperature, and are predicted to experience increasingly extreme weather due to global climate change (Greenville et al. 2012). Temporally and spatially variable flooding rains already are characteristic of arid environments, and these events can result in large numbers of carcasses within arid systems through the drowning of animals (Thibault and Brown 2008; Letnic and Dickman 2010). In addition, animal populations typically experience ‘boom’ periods after significant rainfalls due to pulses in primary productivity (Letnic and Dickman 2006). In turn, these pulses cause changes in assemblage composition by directly altering consumer population numbers and indirectly changing resource availability and the nature of food web interactions (Letnic and Dickman 2010). Although such responses are well documented, there has been no attempt to characterise how scavenger communities respond to large rain events in arid areas, and there is little understanding of how long carcasses persist in arid ecosystems during boom-and-bust periods.

This study examines how vertebrate scavengers respond to a large-scale disturbance event in an Australian desert environment. Specifically, it investigates the impact of a flooding event (that occurred opportunistically during an ongoing study) on vertebrate scavenger activity and community composition, as well as carcass persistence. The study was carried out in both warm and cool seasons and across two contrasting habitats (dune and interdune) before and after the flood, providing a unique opportunity to also examine the influence of these factors on vertebrate scavenging. We predicted that (1) overall activity of vertebrate scavengers would increase post-flood because the flood will attract increased numbers of scavengers from a broader area due to increased resource availability; (2) scavenger composition would shift towards avian scavengers post-flood as the greater mobility of these birds allows faster access to carcasses; and (3) rates of carcass persistence would decrease post-flood due to increased vertebrate scavenging activity. The results provide comprehensive insights into the effects of a flooding event on rates of vertebrate scavenging and carcass persistence rates in an arid setting.

## Materials and methods

### Study site

**Fig. 1:**
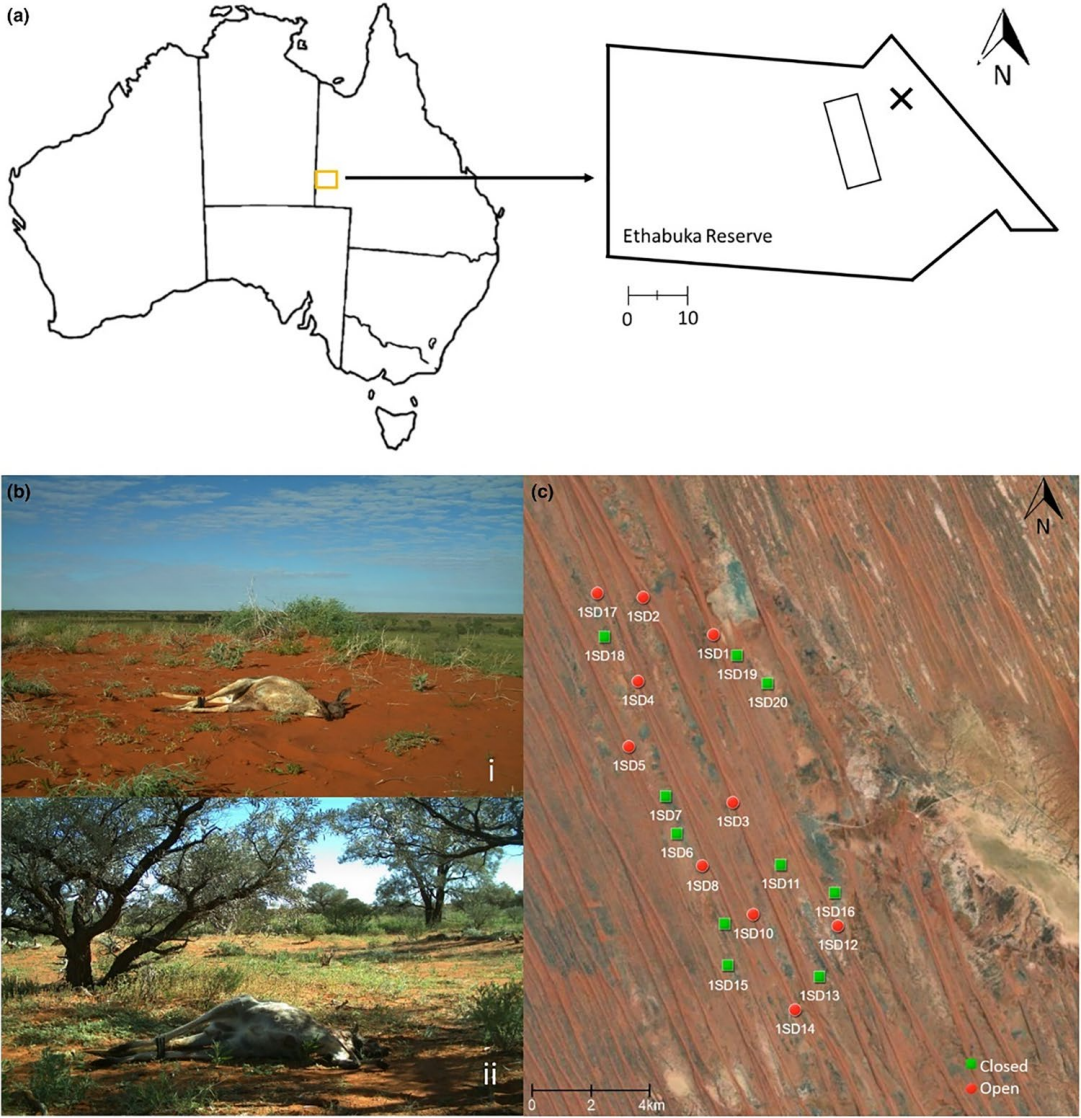
**a** The location of Ethabuka Reserve in the Simpson Desert, Queensland, with the main camp site (23°46′ S, 138°28′ E) marked with a cross and the approximate location of the carcass sites shown by the rectangle. **b** Example of sparsely vegetated dunes (*i*) and stands of gidgee trees in the interdune areas (*ii*) in the Simpson Desert. **c** Location of all the carcasses during the first carcass drop at Ethabuka Reserve. Carcasses were placed in both dune (red circle) and interdune (green square) habitats at least 1 km away from another site during the same carcass drop. A similar configuration was adopted for the subsequent three carcass drops, with all carcasses set at least 200 m from a previous placement (modified after Bragato et al. 2022)

The study was conducted at Ethabuka Reserve in the Simpson Desert, western Queensland, Australia (Fig. 1). Dunefields with parallel, longitudinal sand ridges are the primary landform (Purdie 1984). Vegetation is distinctly zoned, with sand dunes dominated by hummock grassland comprising hard spinifex (*Triodia basedowii*) and dune crests by varied shrubs and cane-grass (*Zygochloa paradoxa*, *Acacia*, *Eremophila* and *Grevillea* species (Letnic and Dickman 2005; Wardle et al. 2015)). Low-lying woodlands and shrublands between the dunes (interdune) host plant communities dominated by *Eucalyptus*, *Acacia* and *Atriplex* species (Wardle et al. 2015). The floristic and structural differences between dune and interdune habitats offered an opportunity to examine habitat effects on scavenging dynamics, with the dune areas having an open canopy and the interdune areas having patches of gidgee (*Acacia georginae*) trees with greater than 20% canopy cover (Fig. 1).

Annual mean maximum temperatures range from 22.8 to 39.6 °C, typical of a hot desert and arid climate (data from Bedourie weather station, 1998–2020, 120 km from Ethabuka Reserve; Bureau of Meteorology 2020). However, the region does experience marked differences in weather between winter and summer, with mean minimum temperatures of 7.6 °C and 25.3 °C, respectively (data from Bedourie weather station, 1998–2020; Bureau of Meteorology 2020). Rainfall is highly variable and fluctuates with season, occurring mostly in the summer months of January to March; the annual average is < 200 mm (data from Bedourie weather station, 1932–2020; Bureau of Meteorology 2020). In 2019, however, Ethabuka Reserve received 154.4 mm of rain in March, with most falling in a single day (149 mm on the 26th March 2019; data from Environdata Pty Ltd weather station, Ethabuka Reserve, 10 km from the study site; courtesy Desert Ecology Research Group) (Figure S1 and S2). This rainfall equalled more than half the total amount of rain that fell that year, and resulted in pooling of water in claypans for at least 2–3 months as well as rapid vegetation growth and flowering of annuals especially *Ptilotus polystachyus* and *Trachymene glaucifolia*. Such localised rain events are rare, and followed 3 months of cyclone-induced heavy rains to the north which caused major river level rises in three channel country catchments (Georgina/Eyre, Diamantina and Thomson/Barcoo/Cooper) near the study site (Bureau of Meteorology 2020). The combination of changes in temperature between winter and summer, and the localised rain and regional flooding, provided an opportunity to investigate how these factors influenced scavenger dynamics and carcass persistence.

### Experimental design

Standardised monitoring of vertebrate scavengers around carcasses was initiated in 2018 as part of an ongoing study. The occurrence of the flood event in April 2019 provided an opportunity to replicate this work after the flood. A total of 80 red kangaroo (*Osphranter rufus*) carcasses were placed in the field over this time period, with 20 placed in the field in June 2018 (cool season) and 20 placed again in October 2018 (warm season) before the flood, and 20 placed in the field in June 2019 (cool season) and 20 placed again in September 2019 (warm season) after the flood. Within each placement, carcasses were distributed equally between dune crests (*n* = 10 carcasses) and interdune (*n* = 10 carcasses) habitats (Fig. 1). Carcasses were located >1 km apart to minimise carcass odours wafting between sites, consistent with previous studies (Cunningham et al. 2018; Spencer et al. 2020). Carcasses weighed on average 25 kg ± 3.6 SD, and average weights were similar for all carcasses drops (average range 24.6–25.6 kg). All carcasses were obtained from a commercial shooter undertaking local culling operations and placed out within 24 h of collection.

To record vertebrate scavenger activity, Reconyx Hyperfire PC800 remote camera traps were positioned 1 m high on a stake about 3 m from the carcass. The cameras were programmed to take ten images per trigger with no delay between triggers (i.e. ‘Rapidfire’) and sensitivity set to high to maximize detection rates when triggered by thermal movement around the carcass. Carcasses were also wired to two 0.45 m stakes that were hammered into the ground, with one stake attached to the neck and the other to the Achille’s tendon of the kangaroo. Attaching the carcasses to the ground ensured that scavengers could be monitored at a single location without a need to move the monitoring camera, but larger scavengers still have the capacity to take parts of the carcass away if desired. This approach is commonly adopted in scavenging studies (Butler and du Toit 2002; Turner et al. 2017). Carcasses were monitored for 30 days from their initial placement in the field.

### Data processing

Images from the remote camera traps were tagged according to visitation events using the photo management software, digiKam (Version 6.4.0). In a visitation event, each photograph was tagged with the species and the maximum number of individuals visiting the carcass. A new event was defined when the time between visits by scavengers was greater than 10 min, following previous scavenging studies in Australia (e.g. Spencer and Newsome 2021) and a separate analysis showing that visits to carcasses by corvids (the most common scavenger) in the study site are generally separated by 5 min or less or greater than 30 min (see Bragato et al. 2022). Data on visitation times were taken from the time stamps on images provided by the cameras. Species were also tagged for their feeding behaviour at each carcass and considered to be scavenging if making oral contact and causing movement or alteration to the carcass. Unidentified species were tagged as unknown species in the data. Australian ravens (*Corvus coronoides*), Torresian crows (*Corvus orru*) and little crows (*Corvus bennetti*) were grouped together as ‘corvids’ due to the challenges of distinguishing between these species in photographs.

The total numbers of scavenging events and total visitation times for each species at the carcasses were calculated. Carcass visitation times were calculated by subtracting the end time of each scavenging event from the start time, and then rounded up to the nearest minute. Carcass persistence was evaluated through camera trap photographs and field observations. The end carcass removal date was defined as when less than 10% of skin and bone carcass biomass was estimated to remain, based on both on-ground inspections and remote camera photos.

### Data analysis

The number of scavenging events and amount of visitation time were analysed in relation to the activity and composition of scavenger assemblages across seasons, habitats and before and after the flood. To standardise and ensure even sampling times, analysis was performed on data from the first 30 days since carcass placement. This covered the main period of vertebrate scavenger activity and when carcasses were typically either fully consumed or rendered to a state of dry decay/skeletonization. Only species documented as scavenging on the carcasses as per the defined criteria were included in the analysis, with herbivores and insectivores excluded.

A series of multivariate analyses was performed in R version 4.0.2 to identify differences in species activity and assemblage composition (“vegan” library package; Oksanen et al. 2023). The event and visitation data were first transformed using a 4th root transformation to ensure that more common species did not over-influence the results. Bray–Curtis dissimilarity matrixes were then generated on the transformed data to produce non-metric multi-dimensional scaling (nMDS) plots and visualise any patterns in assemblages between factor levels. Differences in the numbers of scavenging events and visitation times were tested using permutational multivariate analysis of variance (PERMANOVA), with 999 permutations to calculate *p* values. Given likely differences in scavenging between seasons and habitats before and after the flood, interaction models between these factors were also analysed with visitation events and times as the response variables. Distance-based tests using the betadisper function were carried out to confirm the assumption of homogeneity of multivariate dispersion (“vegan” library package; Oksanen et al. 2023) (Warton et al. 2012). Statistically significant interactions were further examined using PERMANOVAs, with the data split into warm and cool seasons to test separate interactions. The similarity percentages (SIMPER) procedure was conducted on factors that differed to quantify the percentage contribution of each species to the dissimilarities between factors.

To examine carcass persistence, survival analyses using the Cox proportional hazards model were completed (“coxme” library package v 2.2–16; Therneau 2024a). The carcass depletion data were checked to ensure they met the proportional hazards assumptions by visualising the survival curves and performing the Cox proportional hazards test. Season and flood, as factors in the model, were significant (season: *p* = 0.044, flood: *p* < 0.001) and hence in violation of the assumptions. To account for this, the data were stratified into warm and cool seasons. Kaplan–Meir estimates of the survival function and log rank-test results were then plotted in four separate curves comparing dune and interdune habitats and before and after the flood event between warm and cool seasons (library packages: “survival” v3.2–7; Therneau 2024b, “survminer” v0.4.8; Kassambara et al. 2021, and “ggplot2”; Wickham 2016).

## Results

### Scavenging events

**Fig. 2:**
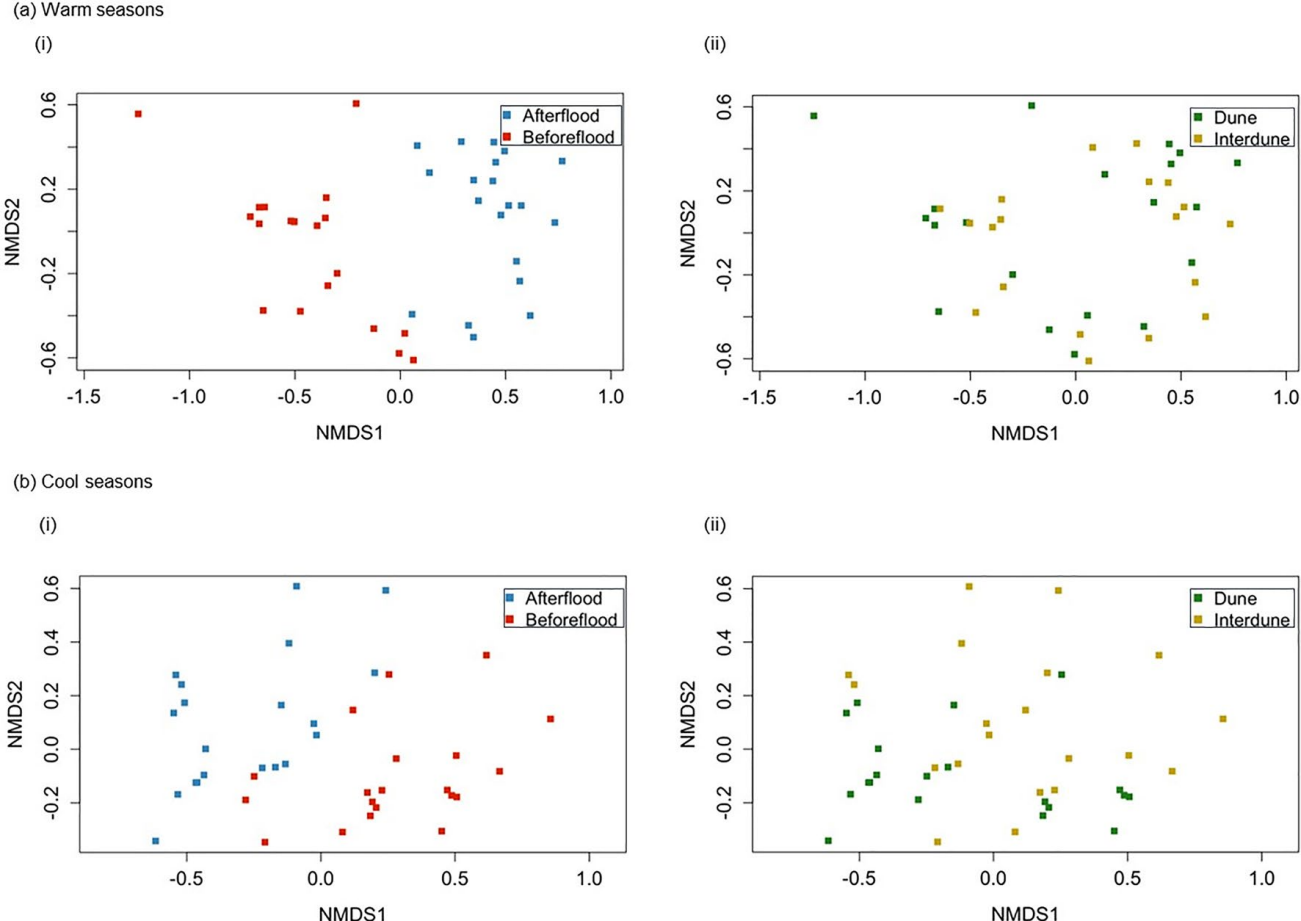
Non-metric multi-dimensional scaling (nMDS) plots based on a Bray–Curtis dissimilarity matrix from the number of scavenger events in **a** warm seasons and **b** cool seasons (*i*) before and after a major flood and (*ii*) between dune and interdune habitats. The arrangement of the data points represents patterns of similarity between species assemblages across the experimental treatments. There is distinct clustering of groups before and after the flood in warm and cool seasons, suggesting there is a difference in species composition pre- and post-flood. Dune and interdune habitat groups are also clustered together in cool seasons. However, there is no clustering in warm seasons between habitats, suggesting no difference in species composition

**Fig. 3:**
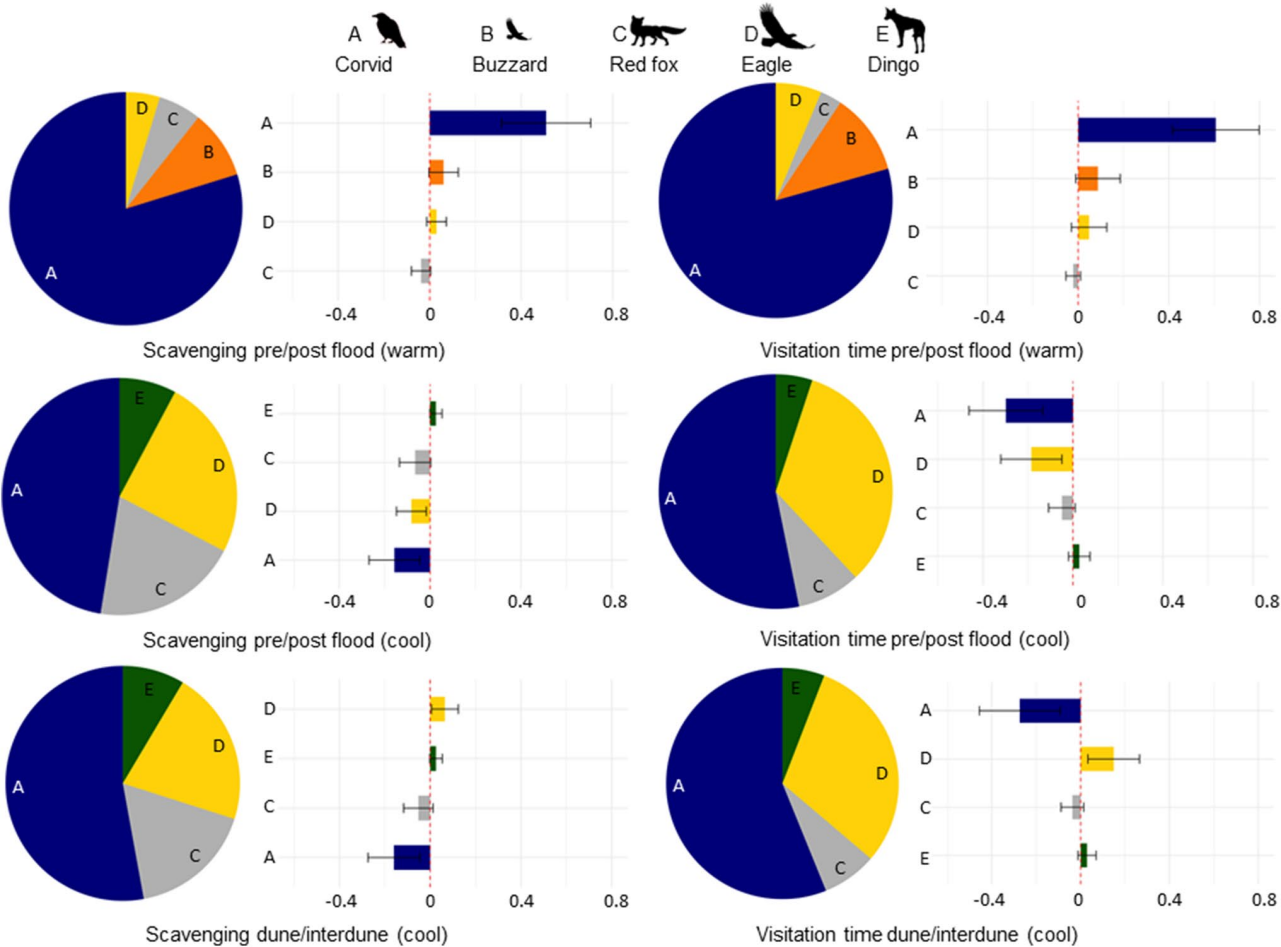
Summary of similarity percentages (SIMPER) outputs (Table S1–S6). Pie charts represent the cumulative sum values for the top 4 species (letters) contributing to differences pre-and post-flood or between habitats (dune/interdune). The associated bar plots represent the average dissimilarity values (±SD). Bar plot values above zero indicate species that increased their use (scavenging) or time (visitation) of carcasses post flood in cool or warm seasons (top four), or increased their use (scavenging) or time (visitation) of carcasses in the interdune area compared to the dune area in the cool season (bottom two), and vice versa for values below zero

There were 8124 scavenging events and 97,976 min of visitation activity recorded of 11 vertebrate scavenger species over the study period. There was a significant interaction between season and habitat, and season and flood (Table 1) for scavenging events at carcasses. The number of scavenging events differed before and after the flood in both the warm seasons (Pseudo *F* = 28.029, *df* = 1, 38, *p* = 0.001; Fig. 2) and the cool seasons (Pseudo *F* = 18.683, *df* = 1, 38, *p* = 0.001; Fig. 2). Corvids made the highest percentage contribution to community-wide differences in the number of scavenging events between warm and cool seasons pre- and post-flood (Fig. 3; Table S1 and S2). In particular, corvid scavenging events quadrupled during the warm season after compared to before the flood. Scavenging events for most other species also increased or remained the same post-flood in the warm season, except for red foxes whose scavenging events declined post-flood (Fig. 3; Table S1). In cool seasons, corvid scavenging events remained high but declined slightly post-flood along with wedge-tailed eagles and red foxes, whereas dingo and black-breasted buzzard scavenging events increased post-flood (Fig. 3; Table S2).

**Table 1:** Permutational multivariate analysis of variance (PERMANOVA) table, testing for differences in the number of scavenger events between the flood event, seasons and habitats

Variables	*df*	Mean squares	Pseudo *F*	*p*
Habitat	1	0.184	3.173	0.025
Season	1	1.013	17.511	0.001
Flood	1	2.451	42.387	0.001
Habitat × season	1	0.236	4.074	0.006
Habitat × flood	1	0.064	1.110	0.346
Season × flood	1	0.493	8.517	0.001
Habitat × season × flood	1	0.053	0.911	0.472
Residuals	72	0.058

There was a significant difference between numbers of scavenging events in dune and interdune habitats in the cool seasons (Pseudo *F* = 3.161, *df* = 1, 38, *p* = 0.029; Fig. 2), but not in the warm seasons (Pseudo *F* = 1.715, *df* = 1, 38, *p* = 0.177; Fig. 2). Differences in the number of scavenging events between habitats in the cool season were attributable mostly to corvids, then wedge-tailed eagles, red foxes and dingoes (Fig. 3; Table S3). Specifically, corvid and red fox scavenging events were higher in dune compared to interdune, whereas wedge-tailed eagle and dingo scavenging events were higher in the interdune compared to dune habitats.

### Visitation times

**Fig. 4:**
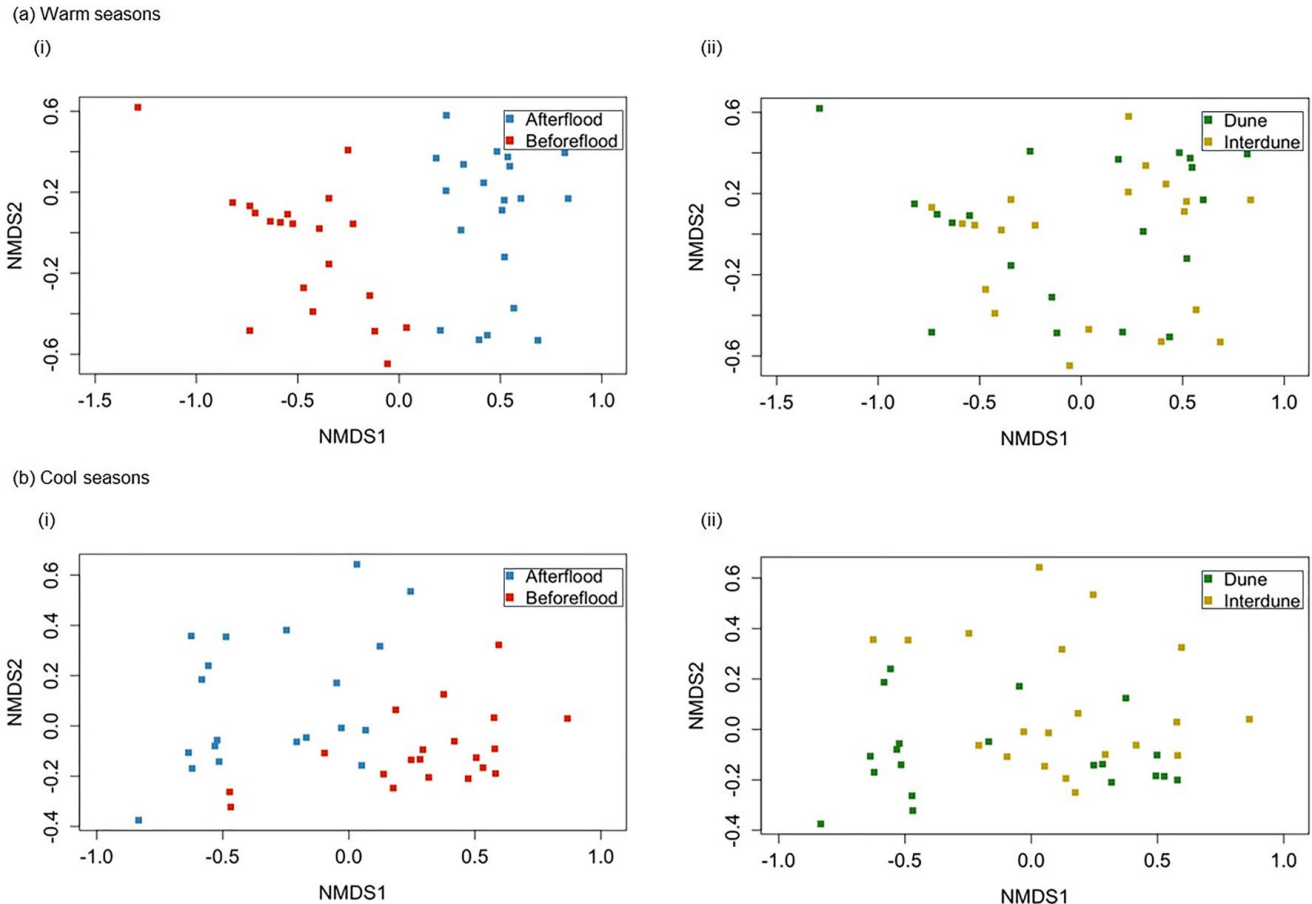
Non-metric multi-dimensional scaling (nMDS) plots based on a Bray–Curtis dissimilarity matrix from scavenger visitation times in **a** warm seasons and **b** cool seasons (*i*) before and after the flood and (*ii*) between dune and interdune habitats. Similar to the number of scavenger events, there is separation and clustering of groups before and after the flood in warm and cool seasons, suggesting differences in species composition pre- and post-flood. Dune and interdune habitat groups are also clustered together in cool seasons, although there is no clustering in warm seasons. This suggests no difference in species composition between habitats in warm seasons

There was a significant interaction between season and flood, and season and habitat (Table 2) for visitation times of scavengers at carcasses. The amount of time scavengers spent visiting carcasses differed before and after the flood in warm seasons (Pseudo *F* = 29.248, *df* = 1, 38, *p* = 0.001; Fig. 3), with this difference mostly attributable to corvids, then black-breasted buzzards, and wedge-tailed eagles who all increased average visitation time after the flood in warm seasons (Fig. 3; Table S4). The visitation times also differed pre- and post-flood in cool seasons (Pseudo *F* = 18.193, *df* = 1, 38, *p* = 0.001; Fig. 4). The scavenger species contributing most to these differences were corvids, wedge-tailed eagles and red foxes who all decreased their visitation times post-flood in the cool season (Fig. 3; Table S5). Comparable to scavenging events, scavenger visitation times differed between dune and interdune habitats in cool seasons (Pseudo *F* = 3.186, *df* = 1, 38, *p* = 0.043; Fig. 4), but not in warm seasons (Pseudo *F* = 1.251, *df* = 1, 38,* p* = 0.277; Fig. 4). Corvids contributed most to the differences in visitation times between habitats in cool seasons, scavenging for longer in dune compared to interdune habitats, whereas wedge-tailed eagles scavenged for longer in interdune habitats (Fig. 3; Table S6).

**Table 2:** Permutational multivariate analysis of variance (PERMANOVA) table testing for differences in the scavenger species visitation times between the flood event, seasons and habitats

Variables	*df*	Mean squares	Pseudo *F*	*p*
Habitat	1	0.210	3.268	0.019
Season	1	1.149	17.854	0.001
Flood	1	2.320	36.046	0.001
Habitat × season	1	0.212	3.294	0.016
Habitat × flood	1	0.077	1.192	0.329
Season × flood	1	1.042	16.190	0.001
Habitat × season × flood	1	0.073	1.137	0.356
Residuals	72	0.064

### Carcass persistence

**Fig. 5:**
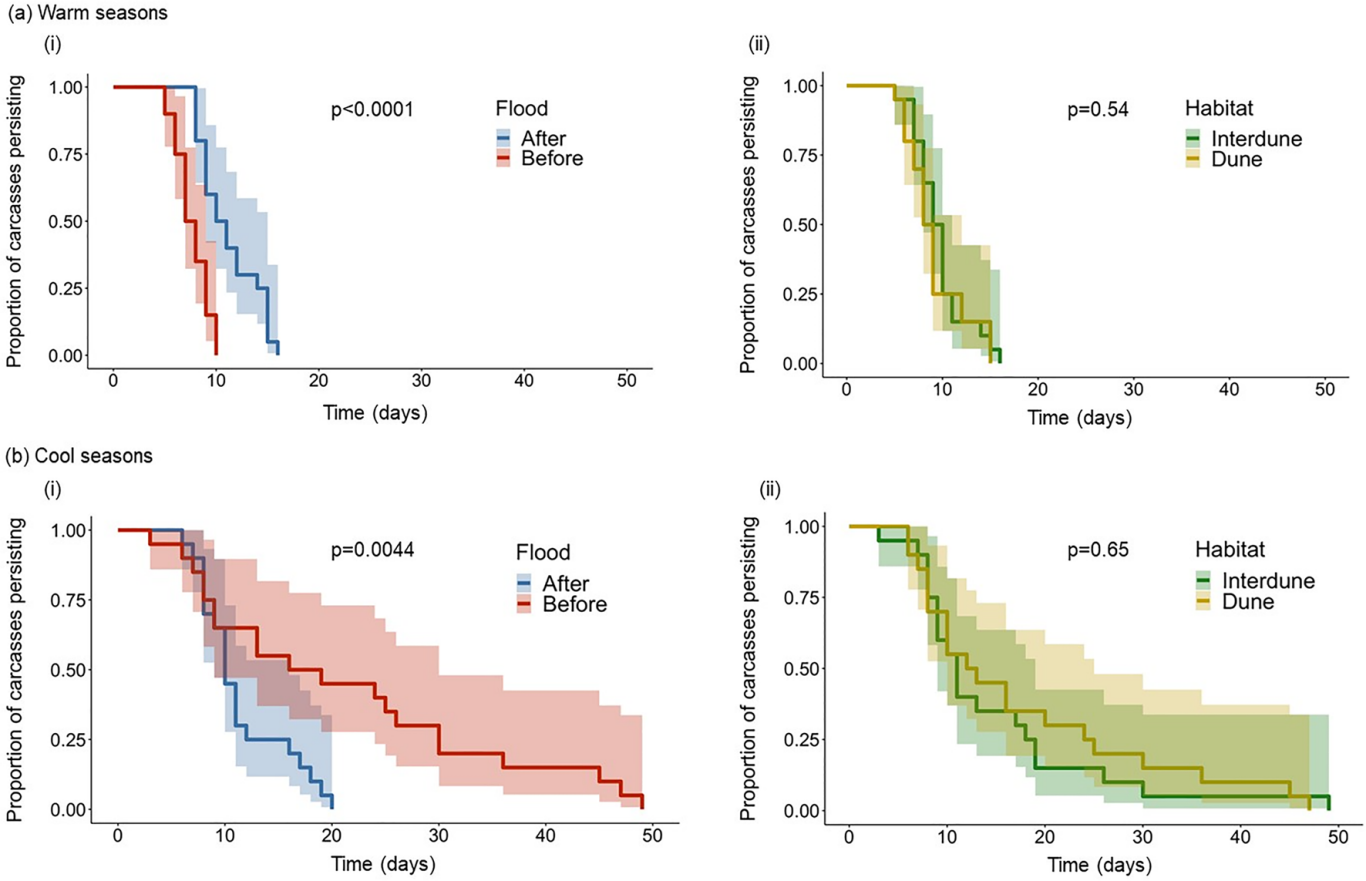
Carcass persistence in **a** warm seasons and **b** cool seasons (*i*) before and after the flood and (*ii*) between dune and interdune habitats. Survival analyses using the Kaplan–Meir estimate with *p* values from the log rank tests. Shading represents 95% confidence intervals. There is no difference in end carcass removal dates between dune and interdune habitats in both warm and cool seasons, however there is a significant difference before and after the flood in both warm and cool seasons

Carcass persistence differed significantly before and after the flood during both warm and cool seasons (Table 3). Carcasses persisted 5.3-fold longer after the flood compared to before the flood in warm seasons (Table 3; Fig. 5). In contrast, carcasses persisted 2.9-fold longer before the flood compared to after the flood in cool seasons (Table 3; Fig. 5). Carcasses persisted to less than 10% of remaining biomass for an average (±SE) of 7.65 ± 0.36 days pre-flood and 11.2 ± 0.63 days post-flood in warm seasons. Rates of carcass loss decreased in cool seasons, with an average (±SE) persistence time of 21.15 ± 3.24 days before and 11.45 ± 0.94 days after the flood. By contrast, carcass survival rates, did not differ between dune and interdune habitat types in either warm or cool seasons (Table 3; Fig. 5). Hazard ratios close to 1 between habitats in warm and cool seasons indicate that carcass persistence was similar in dune and interdune canopy sites (Table 3).

**Table 3:** Cox proportional hazards model table testing differences in the carcass persistence times between the flood event and habitats in warm and cool seasons

Variables	Estimate (β)	Hazards ratios (e^β^)	SE	*z* value	*p*
Habitat (warm)	0.390	1.477	0.332	1.176	0.239
Habitat (cool)	0.046	1.047	0.328	0.140	0.889
Flood (warm)	1.666	5.288	0.413	4.037	<0.001
Flood (cool)	1.071	2.918	0.395	2.711	0.007

## Discussion

This study documented a diverse assemblage of vertebrate scavengers that varied in composition and utilisation of carcass resources between seasons, habitat types and pre- and post-flood. Partially in agreement with the initial predictions, the overall activity of scavengers increased after flooding but only in the warm season, and carcass persistence decreased after the flooding but only in the cool season. Avian scavengers, especially corvids, showed the largest response to the flood with a quadrupling of scavenging events recorded post-flood compared to pre-flood in warm seasons. The results further show that abiotic conditions interact to drive changes in the availability of carcass resources and thus are important in shaping the structure of scavenger communities and ecological processes linked to decomposition more broadly.

### Scavenger activity increased post-flood in warm seasons

The scavenging activity of vertebrates was influenced by season, with the frequency of visitations to carcasses increasing after the flood in warm seasons for most species. However, overall vertebrate scavenging activity decreased in the cool season after the flood, only partially supporting the first prediction. The biological activity of consumer organisms in desert ecosystems is largely associated with the irregular and unpredictable patterns of rainfall (Letnic and Dickman 2006). Eruptions in numbers of animal populations especially small mammals following significant rainfall events are well documented, as a result of bursts in primary productivity (Letnic 2003; Letnic and Dickman 2005; Pavey et al. 2014). The surplus of available resources and associated increases in species abundances, including that of vertebrate scavengers, could have caused the increases in scavenging activity post-flood.

However, pulses in plant productivity after rainfall are potentially lagged, with cooler temperatures especially delaying vegetation growth (Milton et al. 2004). Thus, ecological differences in consumer productivity and subsequent shifts in animal numbers or activity potentially manifest only after longer periods of time. For example, populations of rodent species and their avian predators have been first observed to increase 6 months following rainfall events (Previtali et al. 2009; Pavey and Nano 2013). This provides a possible explanation for increases in scavenger activity in the warm season after flooding, as opposed to in the cool season immediately post-flood. Scavenging activity has also been found previously to decrease regionally where rainfall drops to below-average. For example, feral pigs (*Sus scrofa*) in semi-arid ecosystems were identified scavenging at carcasses only in years when less than half the average annual rainfall was recorded (Brown et al. 2006). Rainfall indirectly regulated the degree of facultative scavenging, with scavengers, like feral pigs, that do not solely rely on carrion as a food resource opting for hunting strategies and alternative foraging opportunities in times of increased prey abundance, and switching to scavenging in times of lower rainfall or drought (Brown et al. 2006; Pereira et al. 2014). Similarly, the extent of carrion consumption by scavengers can increase after winter snows, when resources and live prey are limited (Selva et al. 2005).

### Scavenger composition shifted towards avian scavengers post-flood

Within the scavenger guild, avian scavengers dominated species assemblages, being the most prevalent before and after the flood across both warm and cool seasons. Overall scavenging activity by mammalian species decreased post-flood relative to pre-flood levels and was greatly exceeded by the activity of birds such as corvids, wedge-tailed eagles, and black-breasted buzzards, thus in accordance with the second prediction. These results are consistent with literature demonstrating that corvids, in particular, are often the most abundant scavengers within assemblages, their scavenging activity at carcasses regularly surpassing both mammalian and other avian scavenger species (Read and Wilson 2004; O’Brien et al. 2010; Rees et al. 2020; Newsome and Spencer 2022). Variation in the taxonomic composition of vertebrate scavenger guilds can emerge due to differences in modes of carcass detection between functional groups (Brown et al. 2015). Avian exploitation of carcasses is considered more specialised, using less energy-demanding and time-consuming strategies to search and locate carrion compared to mammalian scavengers (Sebastián-González et al. 2013). Birds are able to detect carcass resources from greater distances and also utilise social cues from other scavengers when foraging (Rösner and Selva 2005; Cortes-Avizanda et al. 2014). Vultures, in particular, regularly capitalise on social information transfer from conspecifics and other scavengers like large raptors (Cortes-Avizanda et al. 2014; Kane et al. 2014). The dominance of avian species within assemblages could therefore be related to their enhanced-scavenging efficiency, in terms of carcass detection. In addition, large bodies of water may accumulate post-flood and restrict access or detection of carcasses, especially by ground-dwelling scavengers. The ability to find and access carcasses from an aerial position may therefore be advantageous and explain the widespread occurrence of avian scavengers at carcasses in this study post-flood.

### Carcass persistence decreased post-flood in cool seasons

Carcass persistence decreased after the flood in cool seasons but increased after the flood in warm seasons, supporting part of our third prediction. Overall carcass biomass loss and removal was, however, much more rapid in warm compared to cool seasons. These trends may be influenced by the abundance and efficiency of the scavenger guilds present. Specifically, apex scavenger species are highly efficient consumers of carcasses and contribute significantly to biomass removal, leading to increased carcass persistence times when they are absent from ecosystems (Olson et al. 2012; Morales-Reyes et al. 2017). For example, carcasses persisted 2.6-fold longer without Tasmanian devils (*Sarcophilus harrisii*) as the top vertebrate predator and scavenger, and the exclusion of vultures resulted in nearly tripled rates of carcass persistence (Ogada et al. 2012; Cunningham et al. 2018). Slight increases in scavenging activity of large vertebrate scavengers, including dingoes in the cool seasons post-flood in our study, provide a potential explanation for the decreases in carcass persistence times we observed, but further assessments of the extent of carcass biomass consumed are needed to fully assess this possibility. In other studies, dingoes have been observed rapidly consuming carcass biomass, and in turn, decreasing carcass persistence times (Spencer and Newsome 2021; Newsome and Spencer 2022).

Higher ambient temperatures also accelerate the process of carcass decay due to increases in decomposition activity of soil microbes and scavenging by invertebrate populations (Carter et al. 2007, 2008; Voss et al. 2009; Matuszewski et al. 2010; Barton et al. 2013; Farwig et al. 2014). Longer rates of carcass persistence in the warm season post-flood were likely caused by the considerable variation in average temperatures during the hotter sampling periods. Indeed, the warm season pre-flood experienced higher average temperatures over the monitoring period when compared to the warm season post-flood (Fig. S2). Heightened microbial and invertebrate activity could have therefore contributed to the reduced carcass persistence pre-flood in the warm season. In addition, our results suggest that increased avian scavenging in the warm period post-flood did little to accelerate carcass biomass loss. This accords with the findings of Newsome and Spencer (2022) who found that increases in avian scavenging post-fire did little to accelerate carcass biomass loss. So, while avian scavengers might increase their rates of scavenging following large-scale disturbance events, there may be little change to carcass persistence unless there is a similar response by mammals, reptiles, or invertebrates.

### Broader implications: evidence of interactive effects

Seasonal and temperature fluctuations are widely accepted to alter scavenging dynamics (Selva et al. 2005; Forsyth et al. 2014; Turner et al. 2017). But, facultative scavenging is also affected by prey availability and susceptibility to predation as well as the amount of animal carcasses deposited naturally in the environment (Pereira et al. 2014). It is therefore plausible that changes in resource abundance across seasons could likewise explain the variations in vertebrate scavenging activity and scavenger composition observed. The structural diversity of habitats is equally important in shaping scavenger communities and thus in determining carcass depletion (Turner et al. 2017; Pardo-Barquín et al. 2019). Substantial differences in scavenging activity and assemblage composition were evident between habitat types, but only in cool seasons. Scavenger guilds in dune habitats were dominated by corvids, likely due to greater ease of carcass detection and accessibility, compared with more densely vegetated areas which can visually obstruct and restrict scavenging by avian species (Bamford et al. 2009; Carrasco-Garcia et al. 2018). Wedge-tailed eagles, however, occurred more in interdune canopy habitats. Disregarding the use of large trees for perching and nesting, this contrasts with current research finding that raptors generally forage in more sparsely vegetated areas with higher visibility (Aumann 2001; Peisley et al. 2017). Dingoes also scavenged slightly more and for longer periods of time in interdune than in dune habitats in cool seasons, similar to species such as black-backed jackals (*Canis mesomelas*), feral pigs and red foxes that have been reported to predominantly utilise carcasses in vegetation-covered locations (Ogada et al. 2012; Carrasco-Garcia et al. 2018).

## Future research directions

**Fig. 6:**
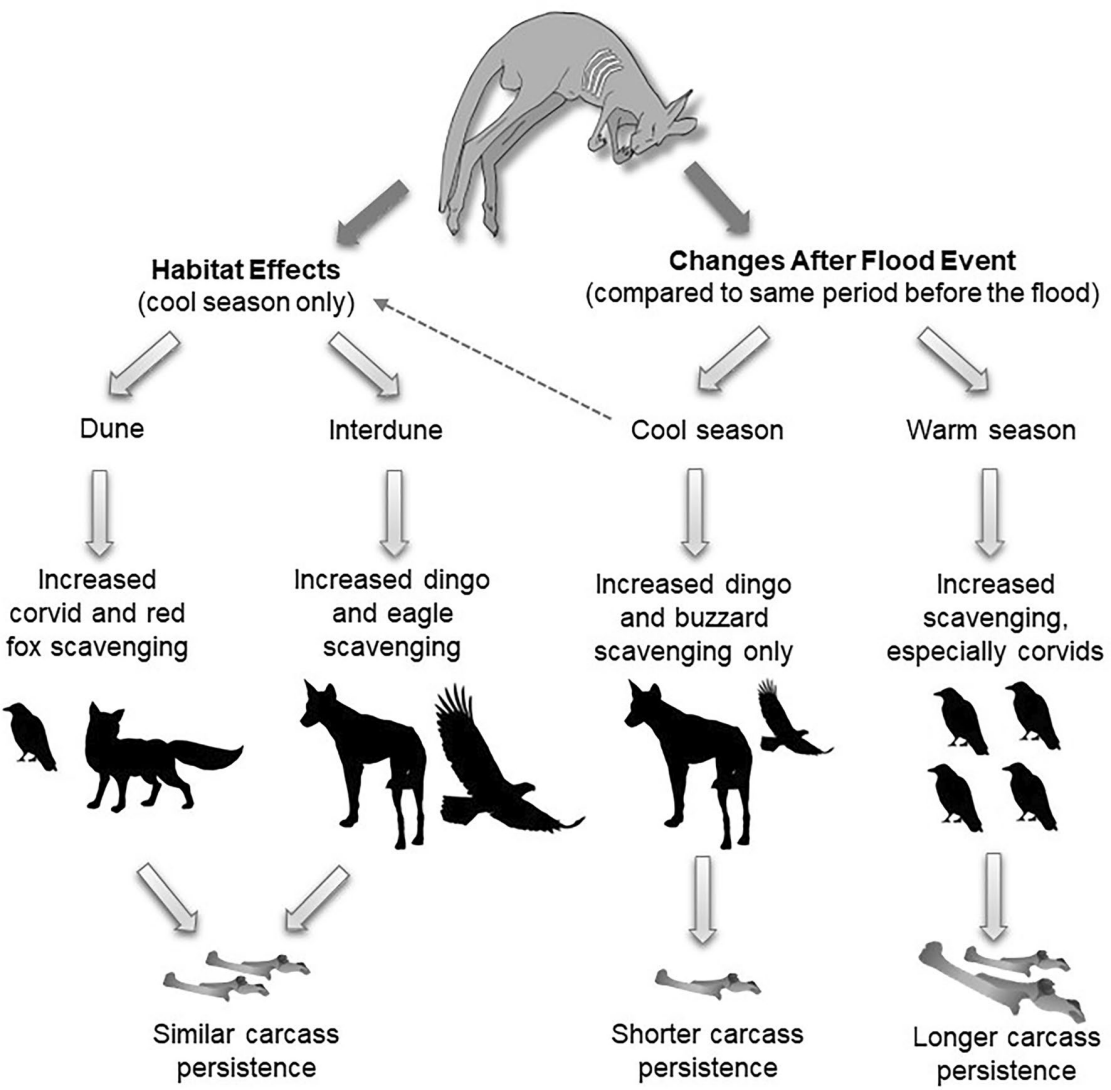
Summary of the main findings. Scavenging rates increased after the flood in the warm season, especially corvids, whereas only dingoes and black-breasted buzzards increased scavenging post-flood in the cool season. Despite increased scavenging in the warm season post-flood, carcasses persisted longer compared to pre-flood, possibly due to cooler temperatures. Habitat affected rates of scavenging in the cool season only, but carcass persistence was the same between dune and interdune canopy habitats

Ultimately, flooding, season and habitat interacted strongly to influence scavenging events and carcass persistence in this study (Fig. 6). There were significant interactions between the effects of season and of flooding and habitat, thus more than one factor simultaneously caused changes in overall scavenger activity, assemblage composition and rates of carcass decay versus one factor independently. The significant differences in pre- and post-flood conditions and the extent to which changes were seen, however, allude to the effects of the flooding event exceeding those of season and habitat and demonstrate that a flood can influence the structure of carrion-based food webs. Further studies are therefore needed to uncover the influence of weather extremes on scavenging dynamics. However, to provide pre- and post-data requires the establishment of ongoing carcass monitoring. Incorporating differences in prey availability, carcass biomass and broader monitoring of scavenger populations including abundances of invertebrate scavenger species would also benefit future studies. This would ultimately increase our understanding of the complex ecological mechanisms driving patterns of scavenging.

### Supplementary Information

Below is the link to the electronic supplementary material.

Supplementary file1 (DOCX 161 KB)

## Data Availability

The datasets used and/or analysed during the current study are available from the corresponding author on reasonable request.

## Code availability

The code used are available from the corresponding author on reasonable request.
