# Supplementary material for: Flooding, season and habitat interact to drive changes in vertebrate scavenging and carcass persistence rates
Source: Oecologia. 2024 Apr 8;204(4):861–74. doi: 10.1007/s00442-024-05531-0 (PMC11062959; doi:10.1007/s00442-024-05531-0)
Supplement: Supplementary file 1 — Supplementary file1 (DOCX 161 KB) [file 442_2024_5531_MOESM1_ESM.docx]

**Supplementary Material**

**
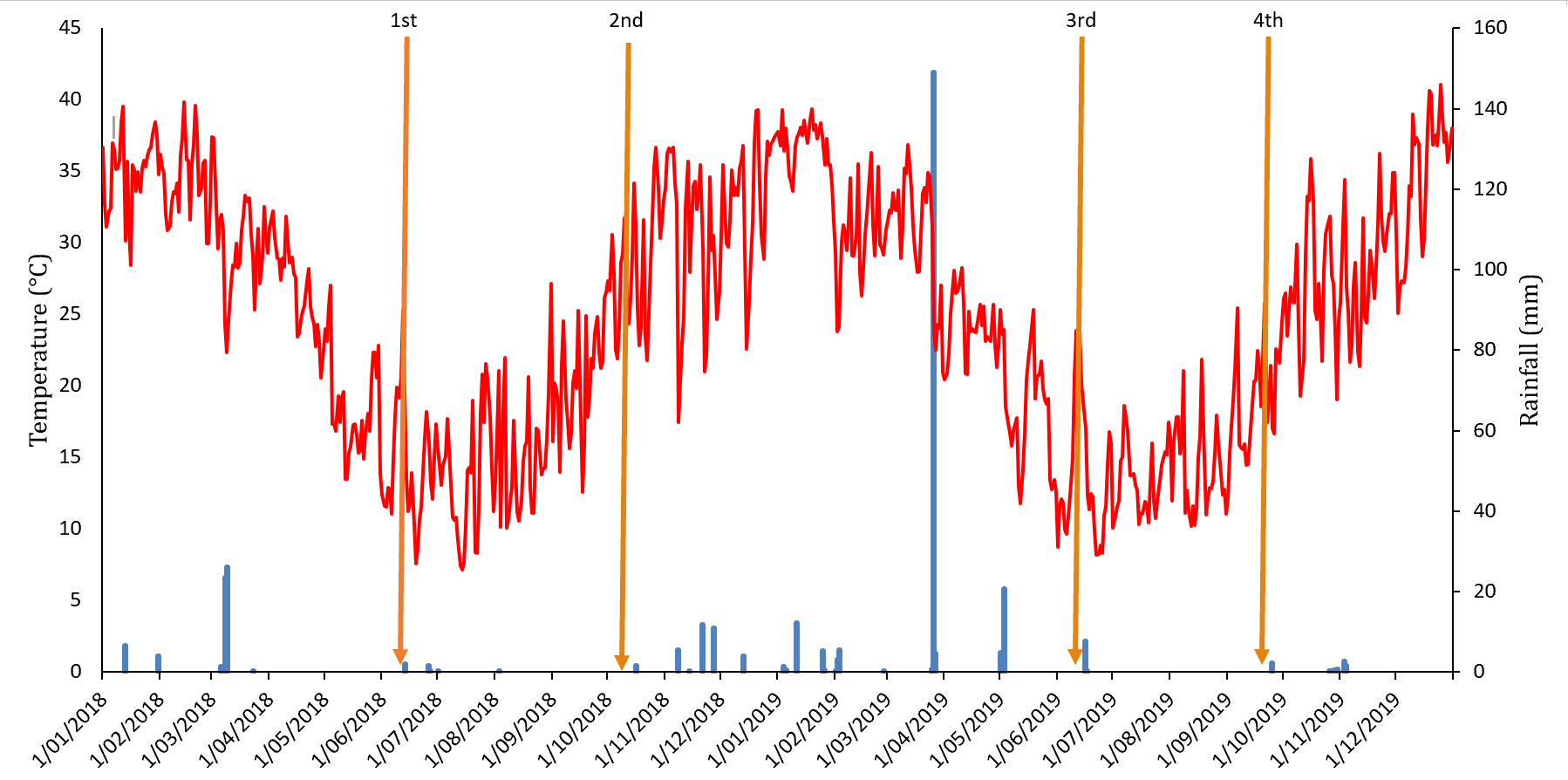
**

**Figure S1**: The daily mean temperature (red line) and daily rainfall (blue columns) measured at Main Camp, Ethabuka Reserve, Simpson Desert. Main Camp is ~10 km from the location of the carcass drops, where data were recorded by an automatic Environdata weather station. Arrows indicate when carcasses were placed in the field. A heavy rainfall event occurred in March 2019, when one year’s average rain fell in one day.

**Figure S2**. Mean daily temperature at Main Camp, Ethabuka Reserve, Simpson Desert, Queensland, during each carcass drop. Main Camp is ~10 km away from the site of the carcass drops, and the data were recorded by an automatic Environdata weather station (Environdata, Warwick, Qld). Average temperatures for across each carcass drop are as follows: 1st = 13.0 °C, 2nd = 29.8 °C, 3rd = 13.7 °C, 4th = 25.4 °C.

**Table. S1** Similarity percentages (SIMPER) table analysing the contribution of species to differences in the number of scavenger events before and after the flood event in warm seasons

| **Species** | **Average dissimilarity** | **sd** | **Average dissimilarity/sd** | **Average Events Before Flood** | **Average Events After Flood** | **Cumulative Sum** |
| --- | --- | --- | --- | --- | --- | --- |
| Corvid (*Corvus coronoides*, *C. orru* and *C. bennetti*) | 0.509 | 0.195 | 2.607 | 24.35 | 100.55 | 0.761 |
| Black-breasted buzzard (*Hamirostra melanosternon*) | 0.061 | 0.064 | 0.957 | 0.00 | 9.50 | 0.852 |
| Red fox (*Vulpes vulpes*) | 0.038 | 0.042 | 0.899 | 5.45 | 0.70 | 0.909 |
| Wedge-tailed eagle (*Aquila audax*) | 0.030 | 0.043 | 0.707 | 1.55 | 4.20 | 0.954 |
| Sand goanna (*Varanus gouldii*) | 0.012 | 0.027 | 0.440 | 0.45 | 2.25 | 0.973 |
| Dingo (*Canis dingo*) | 0.011 | 0.018 | 0.601 | 0.00 | 1.55 | 0.989 |
| Feral cat (*Felis catus*) | 0.003 | 0.004 | 0.695 | 0.10 | 0.45 | 0.993 |
| Black kite (*Milvus migrans*) | 0.003 | 0.007 | 0.361 | 0.00 | 0.50 | 0.997 |
| Magpie (*Cracticus tibicen*) | 0.001 | 0.003 | 0.304 | 0.05 | 0.05 | 0.999 |
| Whistling kite (*Haliastur sphenurus*) | 0.001 | 0.002 | 0.329 | 0.00 | 0.10 | 1.000 |
| Brown falcon (*Falco berigora*) | 0.000 | 0.000 | N/A | 0.00 | 0.00 | 1.000 |

**Table. S2** Similarity percentages (SIMPER) table analysing the contribution of species to differences in the number of scavenger events before and after the flood event in cool seasons

| **Species*** | **Average dissimilarity** | **sd** | **Average dissimilarity/sd** | **Average Events Before Flood** | **Average Events After Flood** | **Cumulative Sum** |
| --- | --- | --- | --- | --- | --- | --- |
| Corvid | 0.157 | 0.112 | 1.402 | 91.25 | 91.15 | 0.441 |
| Wedge-tailed eagle | 0.082 | 0.066 | 1.237 | 22.05 | 2.70 | 0.671 |
| Red fox | 0.066 | 0.069 | 0.959 | 18.35 | 0.00 | 0.857 |
| Dingo | 0.026 | 0.027 | 0.961 | 2.80 | 5.80 | 0.930 |
| Black-breasted buzzard | 0.009 | 0.014 | 0.643 | 0.00 | 2.10 | 0.955 |
| Feral cat | 0.008 | 0.020 | 0.409 | 2.15 | 0.40 | 0.978 |
| Magpie | 0.003 | 0.008 | 0.423 | 0.85 | 0.00 | 0.988 |
| Black kite | 0.002 | 0.004 | 0.518 | 0.25 | 0.30 | 0.994 |
| Brown falcon | 0.001 | 0.002 | 0.450 | 0.25 | 0.05 | 0.997 |
| Whistling kite | 0.001 | 0.004 | 0.259 | 0.05 | 0.15 | 1.000 |
| Sand goanna | 0.000 | 0.000 | N/A | 0.00 | 0.00 | 1.000 |

*Scientific names are given in Table S1.

**Table. S3** Similarity percentages (SIMPER) table analysing the contribution of species to differences in the number of scavenger events between dune and interdune habitats in cool seasons

| **Species*** | **Average dissimilarity** | **sd** | **Average dissimilarity/sd** | **Average Events Dune** | **Average Events Interdune** | **Cumulative Sum** |
| --- | --- | --- | --- | --- | --- | --- |
| Corvid | 0.160 | 0.114 | 1.404 | 96.15 | 86.25 | 0.488 |
| Wedge-tailed eagle | 0.065 | 0.059 | 1.091 | 10.65 | 14.10 | 0.685 |
| Red fox | 0.052 | 0.065 | 0.803 | 9.30 | 9.05 | 0.844 |
| Dingo | 0.026 | 0.028 | 0.918 | 3.45 | 5.15 | 0.923 |
| Black-breasted buzzard | 0.009 | 0.015 | 0.596 | 1.60 | 0.50 | 0.950 |
| Feral cat | 0.008 | 0.019 | 0.427 | 2.45 | 0.10 | 0.976 |
| Magpie | 0.003 | 0.008 | 0.412 | 0.80 | 0.05 | 0.986 |
| Black kite | 0.002 | 0.005 | 0.535 | 0.50 | 0.05 | 0.993 |
| Brown falcon | 0.001 | 0.003 | 0.464 | 0.30 | 0.00 | 0.997 |
| Whistling kite | 0.001 | 0.004 | 0.260 | 0.05 | 0.15 | 1.000 |
| Sand goanna | 0.000 | 0.000 | N/A | 0.00 | 86.25 | 1.000 |

*Scientific names are given in Table S1.

**Table. S4** Similarity percentages (SIMPER) table analysing the contribution of species to differences in the scavenger visitation times before and after the flood event in warm seasons

| **Species*** | **Average dissimilarity** | **sd** | **Average dissimilarity/sd** | **Average Time Before Flood** | **Average Time After Flood** | **Cumulative Sum** |
| --- | --- | --- | --- | --- | --- | --- |
| Corvid | 0.607 | 0.191 | 3.187 | 152.20 | 1266.35 | 0.773 |
| Black-breasted buzzard | 0.087 | 0.098 | 0.892 | 0.00 | 157.90 | 0.883 |
| Wedge-tailed eagle | 0.048 | 0.078 | 0.615 | 13.40 | 91.10 | 0.945 |
| Red fox | 0.022 | 0.032 | 0.702 | 29.30 | 4.10 | 0.973 |
| Dingo | 0.010 | 0.021 | 0.475 | 0.00 | 10.15 | 0.986 |
| Sand goanna | 0.005 | 0.017 | 0.268 | 0.45 | 17.50 | 0.991 |
| Black kite | 0.003 | 0.009 | 0.387 | 0.00 | 8.80 | 0.996 |
| Whistling kite | 0.002 | 0.007 | 0.329 | 0.00 | 4.10 | 0.999 |
| Magpie | 0.001 | 0.003 | 0.239 | 0.05 | 0.40 | 1.000 |
| Feral cat | 0.000 | 0.000 | 0.674 | 0.10 | 0.45 | 1.000 |
| Brown falcon | 0.000 | 0.000 | N/A | 0.00 | 0.00 | 1.000 |

*Scientific names are given in Table S1.

**Table. S5** Similarity percentages (SIMPER) table analysing the contribution of species to differences in the scavenger visitation times before and after the flood event in cool seasons

| **Species*** | **Average dissimilarity** | **sd** | **Average dissimilarity/sd** | **Average Time Before Flood** | **Average Time After Flood** | **Cumulative Sum** |
| --- | --- | --- | --- | --- | --- | --- |
| Corvid | 0.298 | 0.164 | 1.819 | 1373.85 | 664.05 | 0.515 |
| Wedge-tailed eagle | 0.185 | 0.136 | 1.357 | 680.00 | 79.85 | 0.834 |
| Red fox | 0.049 | 0.059 | 0.842 | 163.80 | 0.00 | 0.919 |
| Dingo | 0.028 | 0.048 | 0.591 | 30.50 | 54.15 | 0.968 |
| Black-breasted buzzard | 0.011 | 0.022 | 0.516 | 0.00 | 32.40 | 0.988 |
| Feral cat | 0.003 | 0.010 | 0.337 | 14.10 | 0.40 | 0.994 |
| Black kite | 0.002 | 0.006 | 0.330 | 1.30 | 4.20 | 0.997 |
| Whistling kite | 0.001 | 0.005 | 0.180 | 0.05 | 1.50 | 0.999 |
| Magpie | 0.000 | 0.001 | 0.376 | 1.15 | 0.00 | 0.999 |
| Brown falcon | 0.000 | 0.001 | 0.317 | 1.40 | 0.05 | 1.000 |
| Sand goanna | 0.000 | 0.000 | N/A | 0.00 | 0.00 | 1.000 |

*Scientific names are given in Table S1.

**Table. S6** Similarity percentages (SIMPER) table analysing the contribution of species to differences in the scavenger visitation times between dune and interdune habitats in cool seasons

| **Species*** | **Average dissimilarity** | **sd** | **Average dissimilarity/sd** | **Average Time Dune** | **Average Time Interdune** | **Cumulative Sum** |
| --- | --- | --- | --- | --- | --- | --- |
| Corvid | 0.274 | 0.182 | 1.508 | 1159.55 | 878.35 | 0.537 |
| Wedge-tailed eagle | 0.149 | 0.116 | 1.284 | 327.00 | 432.85 | 0.828 |
| Red fox | 0.037 | 0.051 | 0.726 | 83.15 | 80.65 | 0.901 |
| Dingo | 0.029 | 0.041 | 0.693 | 39.65 | 45.00 | 0.957 |
| Black-breasted buzzard | 0.014 | 0.030 | 0.480 | 22.75 | 9.65 | 0.985 |
| Feral cat | 0.003 | 0.009 | 0.337 | 14.40 | 0.10 | 0.991 |
| Black kite | 0.003 | 0.009 | 0.317 | 5.25 | 0.25 | 0.996 |
| Whistling kite | 0.001 | 0.006 | 0.198 | 0.05 | 1.50 | 0.999 |
| Magpie | 0.000 | 0.001 | 0.355 | 1.10 | 0.05 | 0.999 |
| Brown falcon | 0.000 | 0.001 | 0.325 | 1.45 | 0.00 | 1.000 |
| Sand goanna | 0.000 | 0.000 | N/A | 0.00 | 0.00 | 1.000 |

*Scientific names are given in Table S1.
